# Neuroprotection and Axonal Regeneration Induced by Bone Marrow Mesenchymal Stromal Cells Depend on the Type of Transplant

**DOI:** 10.3389/fcell.2021.772223

**Published:** 2021-11-04

**Authors:** María Norte-Muñoz, Fernando Lucas-Ruiz, Alejandro Gallego-Ortega, David García-Bernal, Francisco J. Valiente-Soriano, Pedro de la Villa, Manuel Vidal-Sanz, Marta Agudo-Barriuso

**Affiliations:** ^1^ Experimental Ophthalmology Group, Instituto Murciano de Investigación Biosanitaria Virgen de la Arrixaca (IMIB-Arrixaca) and Universidad de Murcia, Murcia, Spain; ^2^ Hematopoietic Transplant and Cellular Therapy Unit, Molecular Biology and Immunology Department, Instituto Murciano de Investigación Biosanitaria Virgen de la Arrixaca (IMIB-Arrixaca) and Biochemistry, Universidad de Murcia, Murcia, Spain; ^3^ Systems Biology Department, Faculty of Medicine, University of Alcalá, Alcalá de Henares, Spain

**Keywords:** retinal ganglion cell, optic nerve crush, bone marrow mesenchymal stromal cells, syngraft, allograft, xenograft, neuroprotection, axonal regeneration

## Abstract

Mesenchymal stromal cell (MSC) therapy to treat neurodegenerative diseases has not been as successful as expected in some preclinical studies. Because preclinical research is so diverse, it is difficult to know whether the therapeutic outcome is due to the cell type, the type of transplant or the model of disease. Our aim here was to analyze the effect of the type of transplant on neuroprotection and axonal regeneration, so we tested MSCs from the same niche in the same model of neurodegeneration in the three transplantation settings: xenogeneic, syngeneic and allogeneic. For this, bone marrow mesenchymal stromal cells (BM-MSCs) isolated from healthy human volunteers or C57/BL6 mice were injected into the vitreous body of C57/BL6 mice (xenograft and syngraft) or BALB/c mice (allograft) right after optic nerve axotomy. As controls, vehicle matched groups were done. Retinal anatomy and function were analyzed *in vivo* by optical coherence tomography and electroretinogram, respectively. Survival of vision forming (Brn3a^+^) and non-vision forming (melanopsin^+^) retinal ganglion cells (RGCs) was assessed at 3, 5 and 90 days after the lesion. Regenerative axons were visualized by cholera toxin β anterograde transport. Our data show that grafted BM-MSCs did not integrate in the retina but formed a mesh on top of the ganglion cell layer. The xenotransplant caused retinal edema, detachment and folding, and a significant decrease of functionality compared to the murine transplants. RGC survival and axonal regeneration were significantly higher in the syngrafted retinas than in the other two groups or vehicle controls. Melanopsin^+^RGCs, but not Brn3a^+^RGCs, were also neuroprotected by the xenograft. In conclusion, the type of transplant has an impact on the therapeutic effect of BM-MSCs affecting not only neuronal survival but also the host tissue response. Our data indicate that syngrafts may be more beneficial than allografts and, interestingly, that the type of neuron that is rescued also plays a significant role in the successfulness of the cell therapy.

## Introduction

The attractiveness of mesenchymal stromal cells (MSCs) as an advanced therapy medicinal product (ATMP) lays in their limited antigenicity, anti-inflammatory effects, immunomodulatory properties, and secretion of trophic factors (Le Blanc et al., 2003; Meirelles et al., 2009; Chaudhary et al., 2018; Mishra et al., 2020; Song et al., 2020; Wu et al., 2020; García-Bernal et al., 2021). Of similar importance, they are isolated quite easily from many niches of adult individuals (Kern et al., 2006; Hoogduijn et al., 2014; Valencia et al., 2016; Urrutia et al., 2019), avoiding the ethical problems of embryonic stem cells.

Stem cell therapy for neurodegenerative disorders has two main objectives, neuronal replacement (Coco-Martin et al., 2021) and neuroprotection (Millán-Rivero et al., 2018). In both cases, target reconnection is essential to restore function. Neuronal replacement is a very challenging task still unattainable for patients: circuitry is very complex, neurons are extremely specialized and highly diverse even within the same functional population (Rheaume et al., 2018; Tran et al., 2019). Ameliorating the course of neuronal death and the progression of the disease is a more attainable objective. Thus, although MSCs can differentiate into neurons and glia (Hernández et al., 2020), they are being trialled as neuroprotective ATMPs (Wright et al., 2011; Ng et al., 2014; Uccelli et al., 2019). Ongoing clinical trials involve a wide variety of conditions with different etiologies, such as spinal cord injuries, cerebral stroke, multiple sclerosis, Parkinson’s, Alzheimer’s, autism spectrum, glaucoma, or cerebellar ataxia. In these trials, majority of transplants are allogeneic (www.clinicaltrials.gov).

MSCs from different tissues are being assayed in many preclinical models of neurodegeneration (Zaverucha-do-Valle et al., 2011; Millán-Rivero et al., 2018; Mesentier-Louro et al., 2019; Ahani-Nahayati et al., 2021; da Silva-Junior et al., 2021; Figiel-Dabrowska et al., 2021; Li et al., 2021; Serrenho et al., 2021; Shabanizadeh et al., 2021). The origin of MSCs is crucial because their origin affects their plasticity, immunogenicity and stemness, which in turn will affect their response to *in vitro* amplification and to the host environment. The host species and tissue are also important, because the immune response is species (Zschaler et al., 2014; Webb et al., 2015) and tissue specific (Brown and Esterházy, 2021). Although MSCs have long been thought to be immunologically privileged (Aggarwal and Pittenger, 2005; English et al., 2010; Escacena et al., 2015; Gu et al., 2015), an increasing number of *in vitro* and *in vivo* studies have recently been described that MSCs induce both innate and adaptative host immune responses (Ankrum et al., 2014; Berglund et al., 2017), not only in xenotransplants (Jungwirth et al., 2018) but also in an allogeneic context (Dhingra et al., 2013; Oliveira et al., 2017). Thus, the balance between MSC secretome and MSC immunogenicity could be key for the MSC persistence in the host and in its mediated therapeutic response (Khan and Newsome, 2019).

Since most preclinical studies test human cells in rodents (xenograft) or cells from the same rodent species and strain (syngraft), and majority of clinical treatments are allogeneic, how can we reach translational conclusions based on preclinical experiments? To this, we must add that MSCs from different tissues are tested in different models of neurodegeneration, which are other variables that make it difficult to reach clear conclusions.

Here we purpose to study the effect of the transplant on neuroprotection and regeneration. For this we have grafted human and murine bone marrow-derived MSCs (BM-MSCs) into the vitreous body directly after optic nerve axotomy, a very well characterized model of neuronal degeneration (Sánchez-Migallón et al., 2018b; 2018a). BM-MSCs were chosen because in syngeneic transplants they have neuroprotective and neuroregenerative properties in models of CNS injury (Ankeny et al., 2004; Wright et al., 2011) including optic nerve lesions (Zaverucha-do-Valle et al., 2011; Mesentier-Louro et al., 2019).

## Materials and Methods

### Animal Handling

All animal procedures were approved by the Institutional Animal Care and Use Committee at University of Murcia (Murcia, Spain) and performed according to the guidelines of our Institution (approved protocols A13150201, A1320140704).

Two months old male mice (C57BL/6, BALB/c and C57BL/6-Tg (CAG-EGFP strains) were obtained from the breeding colony of the University of Murcia or purchased from Envigo (Barcelona, Spain) and The Jackson Laboratory (Bar Harbor, ME, United States), respectively. Animals were kept at the University of Murcia animal housing facilities in temperature and light controlled rooms (12 h light/dark cycles) with food and water administered *ad libitum*.

Optic nerve crush, intravitreal injections, OCT and ERG analyses were carried out under general anaesthesia administered intraperitoneally with a mixture of ketamine (60 mg/kg, Ketolar, Parke-Davies, S.L., Barcelona, Spain) and xylazine (10 mg/kg, Rompun, Bayer S.A., Barcelona, Spain). Analgesia was provided by subcutaneous administration of buprenorphine (0.1 mg/kg; Buprex, Buprenorphine 0.3 mg/ml; Schering-Plough, Madrid, Spain). During and after anaesthesia, eyes were covered with an ointment (Tobrex; Alcon S.A., Barcelona, Spain) to prevent corneal desiccation. Animals were sacrificed with an intraperitoneal injection of an overdose of sodium pentobarbital (Dolethal, Vetoquinol; Especialidades Veterinarias, S.A., Alcobendas, Madrid, Spain).

### Experimental Design and Animal Groups

**FIGURE 1:**
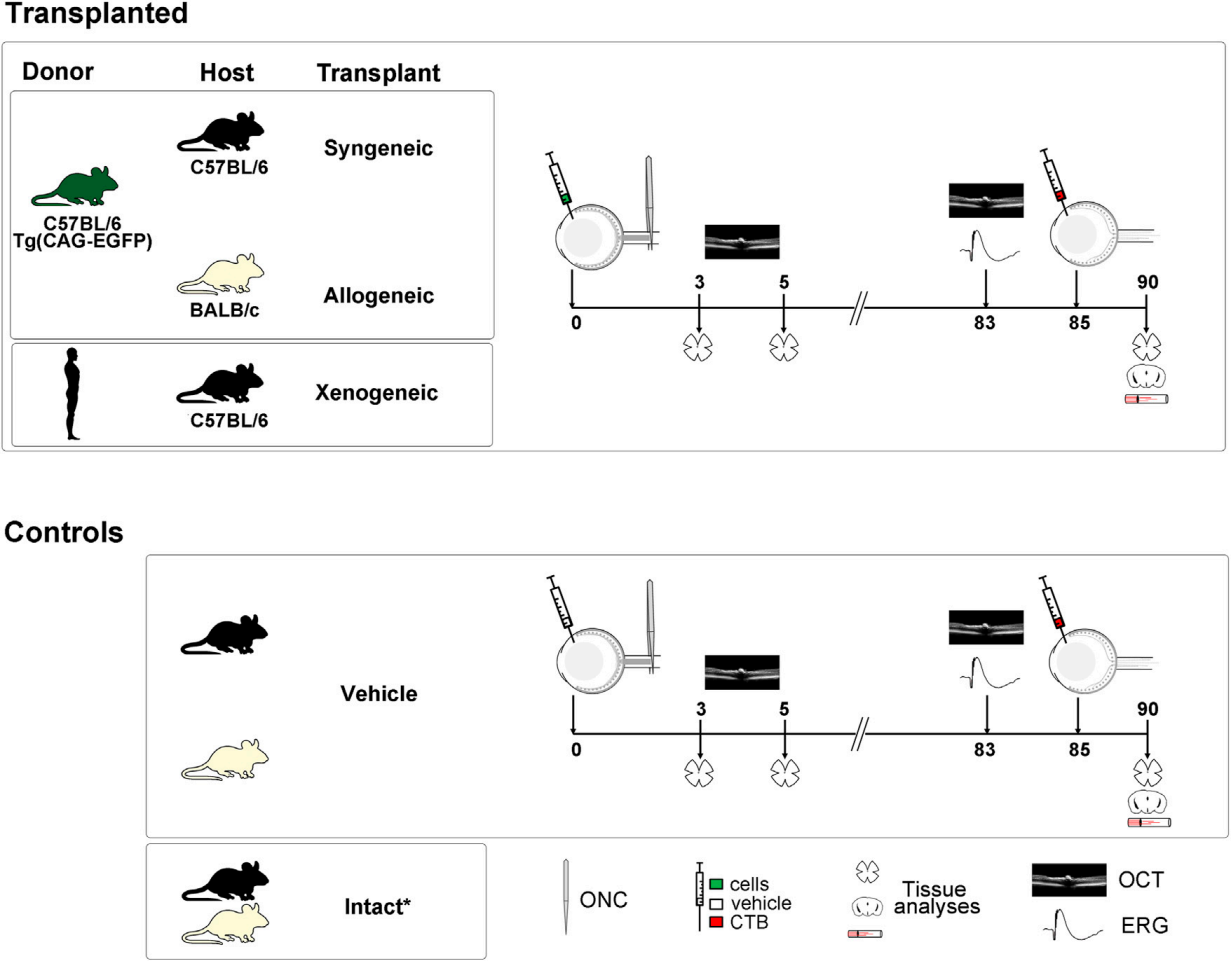
Experimental design. *Controls: intact animals for anatomy and the right eyes for SD-OCT and ERG. Human and dark green mouse silhouettes: donors, Black and off-white mouse silhouettes: recipients and controls. Human and mouse drawings are modified from Vecteezy.com.

See Figure 1. Intact animals were used to assess the total number of RGCs because the undamaged contralateral retinas are not a suitable control (Lucas-Ruiz et al., 2019b). However, because the contralateral effect does not have a significant impact on the long term retinal thickness or functionality after axotomy (unpublished results), the right retinas of the experimental animals were used as control in the electroretinography (ERG) and spectral-domain optical coherence tomography (SD-OCT) analyses. This strategy allows reducing the number of animals because retinal thickness and functionality do decrease with age (Nadal-Nicolás et al., 2018).

### Isolation and Culture of Human and Mouse Bone Marrow Mesenchymal Stem Cells

Human bone marrow samples (hBM) were collected by iliac crest aspiration from 6 healthy volunteers without previous comorbidities (three men and three women, age 21–45 years old) after written informed consent and after the approval of the local Ethics Committee of the University Hospital Virgen de la Arrixaca (HUSA19/1531.February 17, 2020). Bone marrow was collected in syringes containing 20 U/ml sodium heparin followed by a Ficoll-Paque density gradient separation by centrifugation at 470 g for 30 min at R/T. Thereafter, mononuclear cell fraction was collected, rinsed twice with phosphate buffered saline (PBS) (Merck Life Science S.L.U. Madrid, Spain) and seeded into 75-cm^2^ culture flasks (Merck Life Science) at 1.6 × 10^5^ cells/cm^2^ in Minimum Essential Medium Eagle (Thermo Fisher Scientific, Madrid, Spain) supplemented with 10% fetal bovine serum (FBS) (BioWhittaker, Walkersville, MA, United States), 1% penicillin/streptomycin (P/S) (Thermo Fisher Scientific) and 1% L-glutamine (Merck Life Science). After 3 days of culture at 37°C and 5% CO_2_, unattached cells were removed and fresh culture medium was added and replaced twice a week.

Mouse bone marrow mesenchymal stem cells (mBM-MSCs) were isolated from *β*-actin-GFP transgenic C57BL/6-Tg (CAG-EGFP) (The Jackson Laboratory). Briefly, mice aged 6–8 weeks were euthanized by cervical dislocation and tibias and femurs were collected and washed with PBS containing 1% P/S. Then, bone epiphyses were excised and bone marrow was flushed out using a 25-gauge needle and syringe containing low glucose Dulbecco’s Modified Eagle’s Medium (DMEM) (Thermo Fisher Scientific). After two washing steps with PBS, BM cells were seeded into 75-cm^2^ culture flasks at 1.6 × 10^5^ cells/cm^2^ and cultured in low glucose DMEM medium containing 15% FBS, 1% P/S and 1% L-glutamine following the same protocol as for human cells. When cultures were 70–80% confluent, human and mouse BM-MSCs were subcultured at 5 × 10^3^ cells/cm^2^ and used in passages 3–4 for subsequent experiments. Human and mouse BM-MSCs were immunophenotypically characterized by flow cytometry (FACS Canto II, BD Biosciences, San Jose, CA, United States) as previously described (Millán-Rivero et al., 2019; García-Bernal et al., 2020).

### Optic Nerve Crush

The left optic nerve was crushed at 0.5 mm from the optic disc following previously described methods (Galindo-Romero et al., 2013b; Sánchez-Migallón et al., 2018b). In brief, to access the optic nerve at the back of the eye, an incision was made in the skin overlying the superior orbital rim, the supero-external orbital contents were dissected, and the superior and external rectus muscles were sectioned. Then, the optic nerve was crushed for 10 s using watchmaker’s forceps. Before and after the procedure, the eye fundus was observed through the operating microscope to assess the integrity of the retinal blood flow.

### Intravitreal Injections

All intravitreal injections were done in a final volume of 2.5 μl following previously published methods (Galindo-Romero et al., 2013b; Sánchez-Migallón et al., 2018b; Lucas-Ruiz et al., 2019c). BM-MSCs were resuspended and administered in DMEM medium at a concentration of 8 × 10^3^ cells/µL, and other groups injected with DMEM alone were used as vehicle controls. Intravitreal administration of the *β* subunit of the cholera toxin (CTB) coupled to Alexa Fluor 555 (Invitrogen, Thermofisher, Madrid Spain) was used to anterogradely trace RGC axons.

### Electroretinography

Full-field ERG was performed as described elsewhere (Alarcón-Martínez et al., 2010; Valiente-Soriano et al., 2019). Briefly, initially scotopic ERG waves were recorded binocularly from anaesthetised dark-adapted mice in response to a stimulus intensity of -4.3 (Scotopic Threshold Response), -2.5 (Rod Responseand 0.5 log cd·s/m^2^ from a Ganzfeld dome that provided illumination of the whole retina. For the photopic study of electroretinographic waves, the animals were adapted to the light for 5 min and a background light of 30 cd/m^2^ was used throughout the recording. Scotopic and photopic responses were recorded using Burian-Allen corneal bipolar electrodes simultaneously in both eyes. A drop of methylcellulose (Methocel 2%^®^; Novartis Laboratories CIBA Vision, Annonay, France) was used between the cornea and the electrodes to improve signal conductivity. The reference electrode was placed in the mouth and a needle at the base of the tail was used as a ground electrode. The electrical signals were digitized at 20 KHz using a Power Lab data acquisition board (AD Instruments, Chalgrove, United Kingdom). Standard ERG waves were analysed according to the International Society for Clinical Electrophysiology of Vision (ISCEV). For each wave, the implicit time was measured at the peak of the maximum response.

### Spectral Domain-Optical Coherence Tomography

Both retinas were analyzed under SD-OCT (Spectralis; Heidelberg Engineering, Heidelberg, Germany) adapted with a commercially available 78-D double aspheric fundus lens (Volk Optical, Inc., Mentor, OH, United States) mounted in front of the camera unit as described previously (Rovere et al., 2015; Valiente-Soriano et al., 2019). After anaesthesia, a drop of 1% tropicamide (Alcon-Cusí, S.A. Barcelona, Spain) was instilled in both eyes to induce mydriasis. Eyes were carefully kept hydrated with artificial tears and a custom-made contact permeable lens was placed on the cornea to maintain corneal hydration and clarity. Imaging was performed with a proprietary software package (Eye Explorer, version 3.2.1.0; Heidelberg Engineering). Retinas were imaged using a raster scan of 31 equally spaced horizontal B-scan. Thickness of the total, inner and outer retina was measured manually close to the optic nerve head and at 1 mm from it always in central sections spanning the optic disc. Volume of the central retina was calculated by the software after manually aligning the inner and outer retinal limits. Finally, mBM-MSC-GFP cells were visualized in vitreous with the blue light autofluorescence (BAF) mode of the OCT.

### Tissue Processing

Animals were perfused transcardially with 0.9% saline solution followed by 4% paraformaldehyde in 0.1 M phosphate buffer. Retinas were prepared as flat mounts (Galindo-Romero et al., 2011). Brains were cryoprotected in increasing solutions of sucrose, embedded in Tissue-Tek (Sakura, Sakura-Finetek, Barcelona, Spain) and cryostated at 25 µm. Optic nerves were cleared using the CUBIC protocol (Susaki et al., 2014). Briefly, after washing the nerves in PBS, they were kept in scale 1 solution (Susaki et al., 2015; Liang et al., 2016) at 37°C for 4 days. Nerves were mounted in the same solution for imaging.

### Immunodetection

Immunodetection in flat mounts and brain coronal sections was carried out as reported (Galindo-Romero et al., 2011; Nadal-Nicolás et al., 2015). Primary antibodies were: mouse anti-Brn3a (1:500; MAB1585, Merck Millipore; Madrid, Spain), mouse anti-human mitochondria (1:800, ab3298 Abcam, Cambridge, United Kingdom), and rabbit anti-melanopsin (1:1,000; AB-N39 Advanced Targeting Systems ATS, Joure, Netherlands). Secondary detection was carried out with Alexa Fluor-coupled secondary antibodies (1:500; Molecular Probes; Thermo Fisher Scientific, Madrid, Spain). Retinal whole-mounts and brain coronal sections were mounted with anti-fading mounting media.

### Image Acquisition and Analyses

Images were acquired using a Leica DM6B epifluorescence microscope (Leica Microsystems, Wetzlar, Germany). Retinal photomontages were reconstructed from individual squared images of 500 μm. Brn3a^+^RGCs were quantified automatically and m^+^RGCs manually dotted on the photomontages and then quantified. RGC distribution was assessed by isodensity or neighbour maps using previously reported methods (Galindo-Romero et al., 2011; Lucas-Ruiz et al., 2019b). In brief, isodensity maps show the density of RGCs with a colour scale that goes from 0–500 RGCs/mm^2^ (purple) to ≥3,200 RGCs/mm^2^ (red). Those maps are useful to visualize the distribution of abundant cell populations. However, to assess the topography of low number populations (i.e., m^+^RGCs or the number of surviving RGCs long-term after axotomy) neighbour maps are better suited because they depict the number of neighbours around a given cell in a radius of 0.2 mm with a colour scale that goes from 0–2 neighbours (purple) to >21 neighbours (dark red).

### Statistical Analyses

Data were analyzed and plotted with GraphPad Prism v.7 (GraphPad, San Diego, CA, United States). Data are presented as mean ± standard deviation (SD). Differences were considered significant when *p* < 0.05. Statistical tests and number of analyzed samples are detailed in results.

## Results

### Immunophenotypic Characterization of Human and Mouse Bone Marrow-Mesenchymal Stromal Cells

**FIGURE 2:**
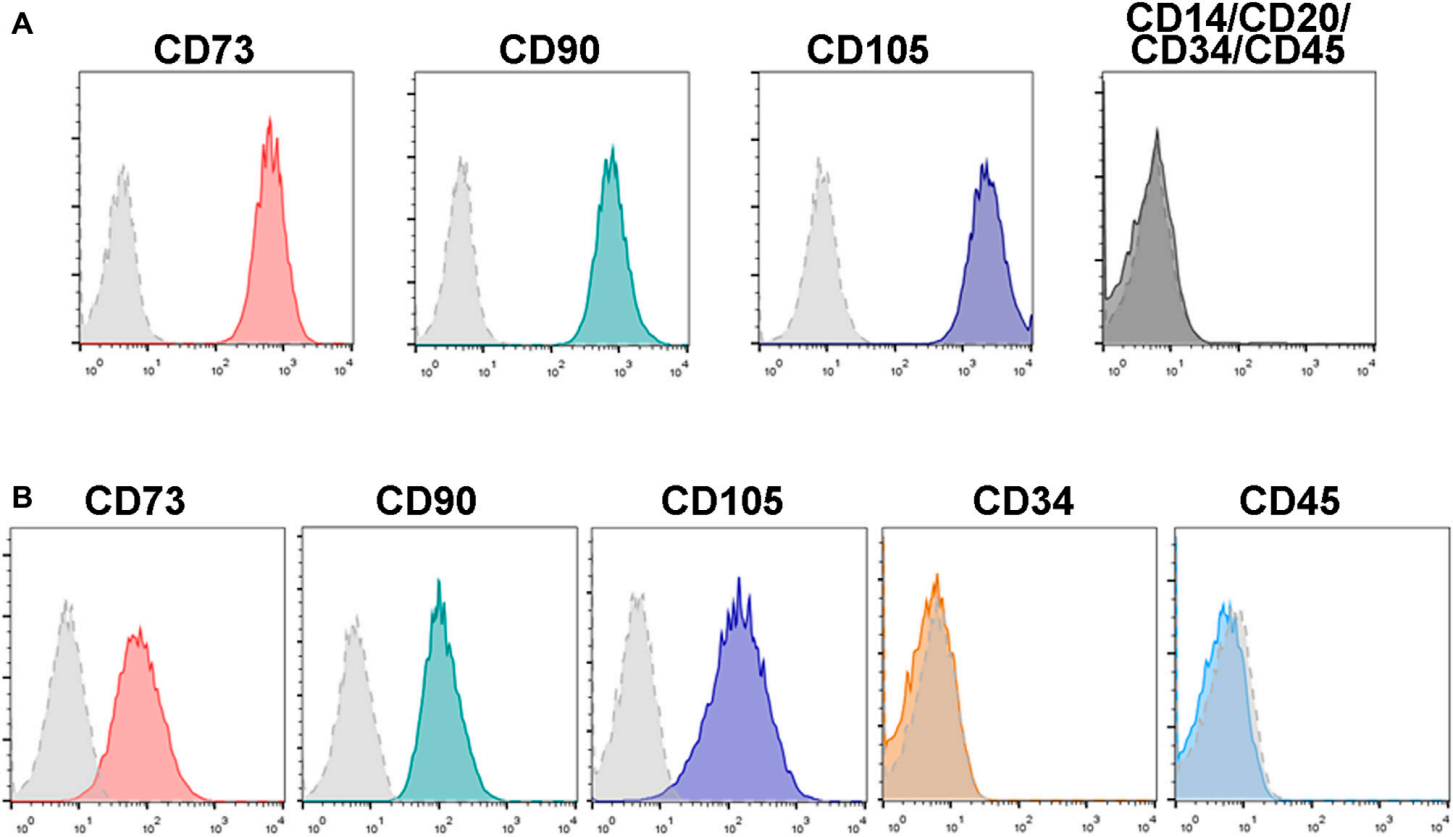
Immunophenotypical analysis of human and mouse BM-MSCs. MSCs isolated from human **(A)** and mouse **(B)** bone marrow express typical MSC markers such as CD73, CD90 and CD105, whereas expression of the hematopoietic markers CD14, CD20, CD34, and CD45 are low or negative. Control isotype antibodies staining are shown as dotted light grey histograms. Histograms show representative flow cytometry results obtained from *n* = 3 separate human and mouse BM-MSC samples.

After isolation, both human and mouse BM-MSCs displayed a spindle-shaped fibroblastic morphology in culture. Flow cytometry immunophenotyping analyses showed that human and mouse BM-MSCs express high levels of the mesenchymal markers CD73, CD90 and CD105, and negligible expression of typical hematopoietic markers such as CD14, CD20, CD34 and CD45 (Figure 2).

### Human and Mouse Bone Marrow-Mesenchymal Stromal Cells Survive in the Vitreous up to 5 days After Transplantation

**FIGURE 3:**
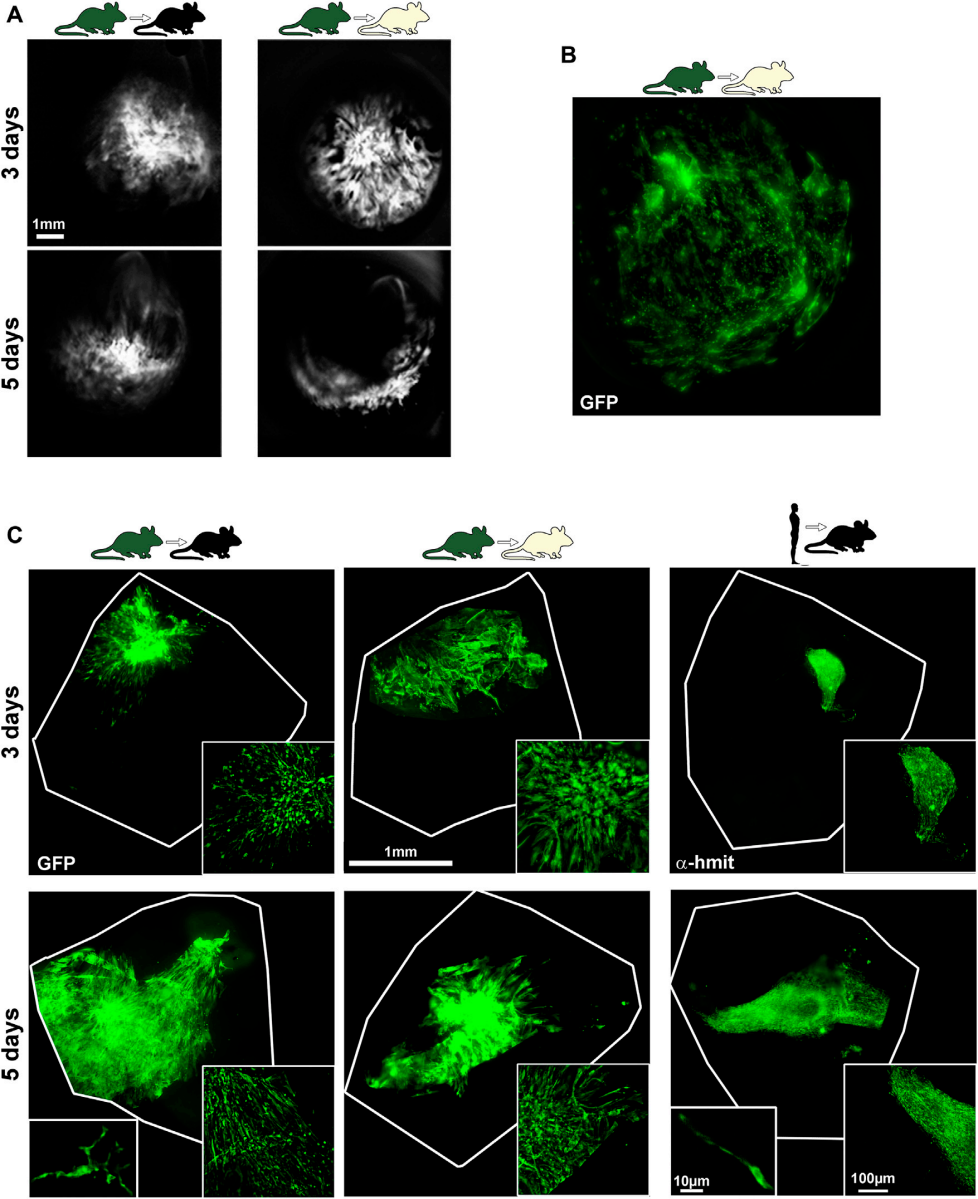
Visualization of BM-MSCs. **(A)**: *In vivo* OCT images showing mBM-MSCs in the vitreous body at 3 and 5 days after syngeneic (left) and allogeneic (right) transplants. **(B,C)**: *Ex vivo*, mBM-MSCs were observed attached to the lens [**(B)**, allogeneic transplant] and on the retinal surface forming a mesh **(C)**. In **(C)**, the retinal petals where cells were injected are outlined. At the bottom right of each image is shown a magnification of the BM-MSC meshes. At the bottom left of 5 days syngraft and xenograft panels are shown high power magnifications of individual mouse and human BM-MSCs, respectively. Grafts were observed at 3 and 5, but not at 90 days. α-h-mit: anti-human mitochondrial immunostaining.

mBM-MSCs-GFP^+^ were observed *in vivo* in the vitreous using the BAF mode of the SD-OCT (Figure 3A). Analyses in flat mounts showed that the cells did not integrate in the retina. Instead, BM-MSCs were attached to the lens (Figure 3B) and forming a mesh on top of the ganglion cell layer that was visible at 3 and 5 but not at 90 days (Figure 3C). After transplantation, human and mouse BM-MSCs displayed different morphologies: mBM-MSCs showed a branched structure, while hBM-MSCs retained the spindle-shaped morphology observed in culture. Finally, hMSCs formed tighter and more compact meshes than mBM-MSCs (Figure 3C, magnifications).

### Bone Marrow-Mesenchymal Stromal Cells Xenogeneic Transplant Alters the Retinal Structure

**FIGURE 4:**
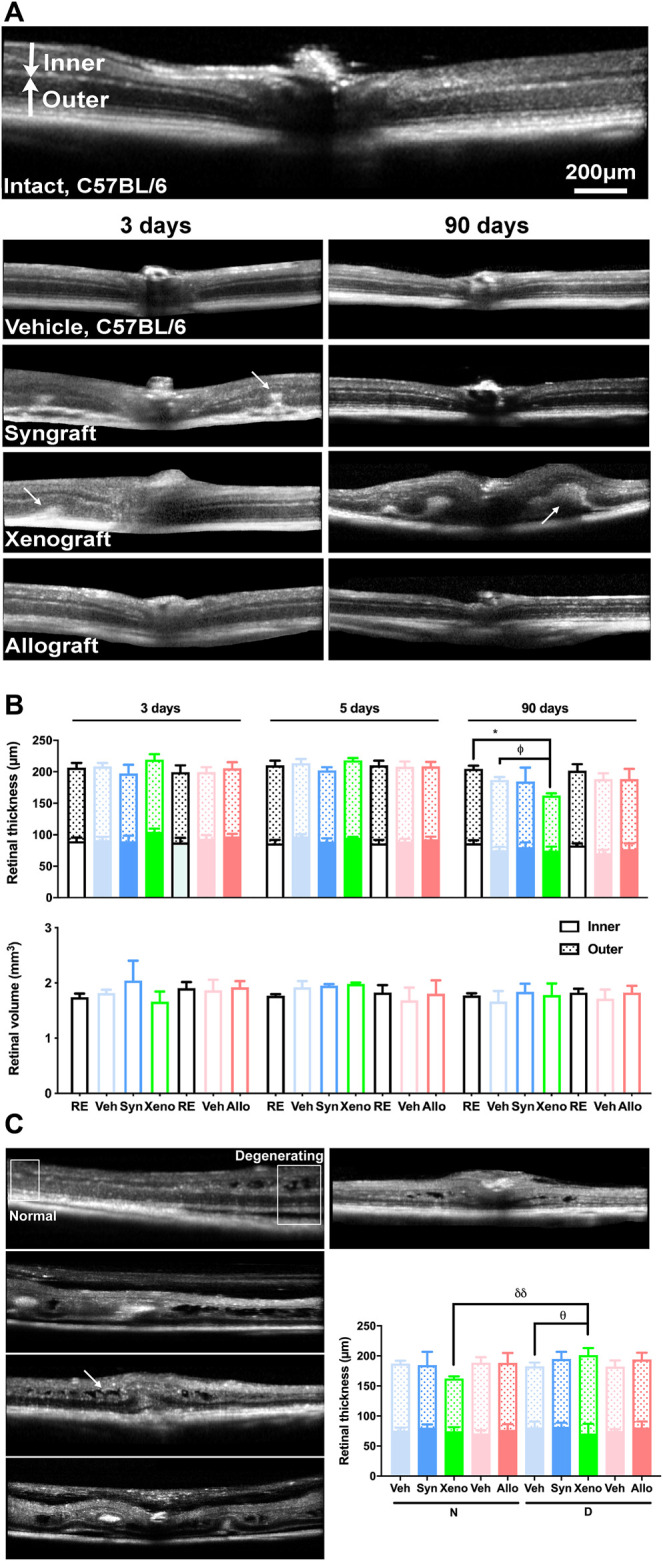
Xenotransplant causes retinal edema, detachment, and thinning. **(A)**: *In vivo* OCT sections spanning the optic nerve in control (RE, 5 months old mouse), vehicle, and transplanted retinas analyzed at 3 or 90 days post-ONC. Intact and vehicle images are from C57/Bl6 mice because there was no difference between the recipient strains. In syngrafted and xenotransplanted retinas there were hyper-reflective areas below the retina (arrows) observed at 3 days that disappeared at 90 days in the syngeneic transplant but remained and grew in the xenotransplant causing edemas, subretinal fluid, retinal folding, and detachment (arrows). **(B)**: Top, stacked column graphs showing the total, inner and outer retinal thickness ± SD (µm) measured at 1 mm from the optic disc in central sections. Bottom, column graphs showing the central retinal volume ± SD (mm^3^) in control and experimental animals. Xenografted retinas were significantly thinner than their right contralateral and vehicle-treated ones (*p < 0.05, compared to right eyes; ^θ^ *p* < 0.05, compared to vehicle. Kruskal-Wallis test, Dunn’s multiple comparisons test) however their retinal volume did not diminish. **(C)**: *In vivo* SD-OCT sections from the central retina of 90 days xenografted animals showing different abnormalities (arrow points to retinal edemas). Framed areas in the top left image show non-degenerating (apparently normal/healthy) and degenerating regions. Because the normal areas were thinner and the degenerating areas were thicker (quantification in graph), the retinal volume did not change (^θ^ *p* < 0.05, compared to its vehicle; ^δδ^ *p* < 0.01, comparing normal and degenerating areas. Non-parametric Mann-Whitney test). *n* = 5 animals/group/time point at 3 and 5 days, and *n* = 4/group at 90 days.

In the OCT sections of syngeneic and xenografted animals analyzed at 3 and 5 days there were hyper-reflective areas below the retina that disappeared in the syngeneic group, but progressed in the xenogeneic one, causing retinal detachment, edema and folding (Figure 4A, arrows). These anomalies were found mainly around or near the optic nerve head.

Retinal thickness and volume were similar between strains and did not change at 3 and 5 days in any of the experimental groups or at 90 days in the allografted and syngrafted groups. As for the xenografted retinas, they significantly thinned at 90 days, but nevertheless their volume remained within normal values (Figure 4B). Retinal thickness was measured at 1 mm from the optic disc, where the retinal structure was, normally, well preserved. Would volume maintenance be related to significant swelling in areas of degeneration that compensates for thinning? We measured the retinal thickness in the degenerating areas, which were usually located near the optic disc and found that indeed the xenografted retinas were significantly thicker than the rest of the groups (Figure 4C).

### Syngeneic Transplant of Bone Marrow-Mesenchymal Stromal Cells Neuroprotects Both Functional Subtypes of Retinal ganglion cells

**FIGURE 5:**
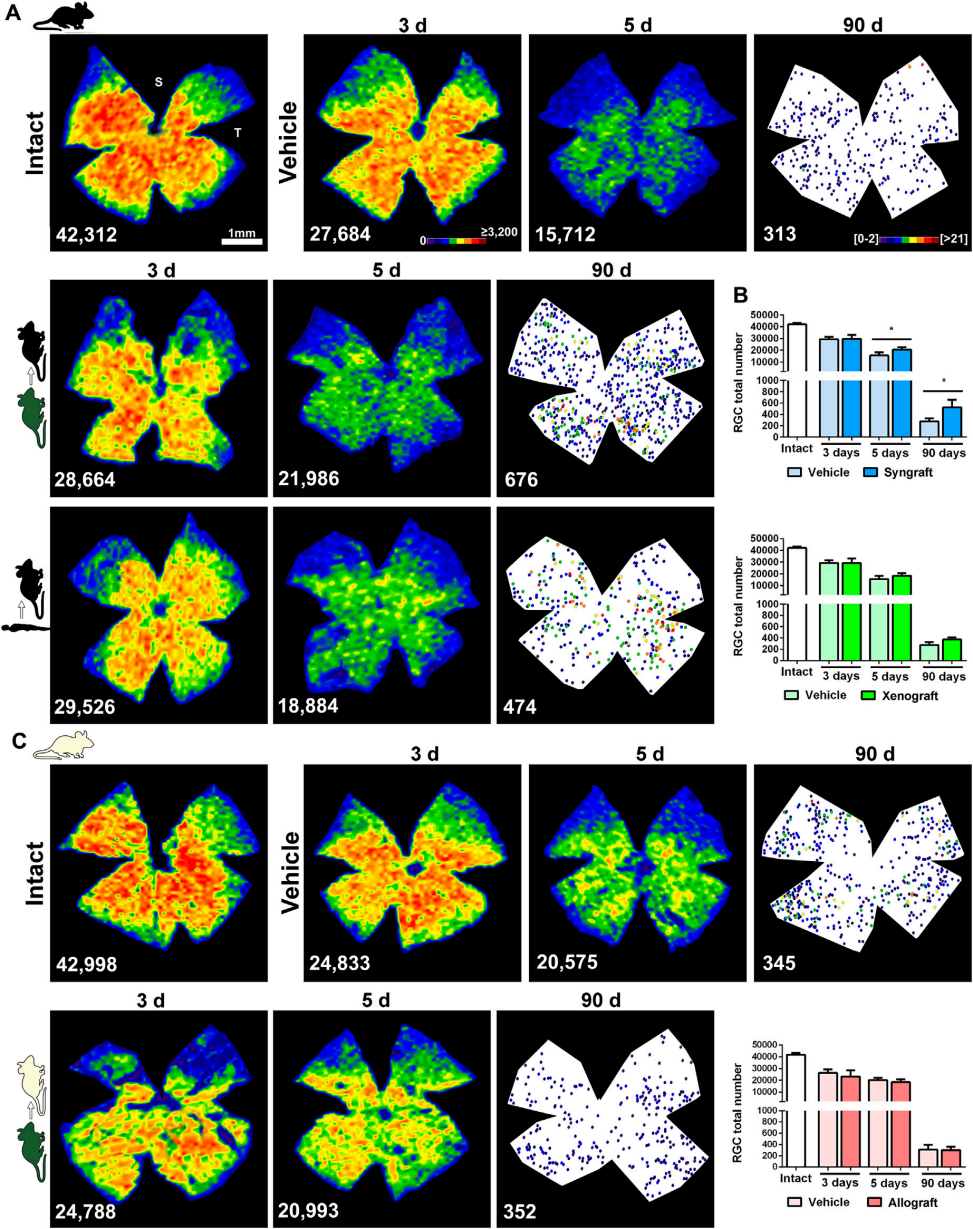
Syngeneic transplant neuroprotects vision-forming RGCs. **(A)**: Representative isodensity maps (intact and 3 and 5 days after ONC) or neighbour maps (90 days after ONC) showing the distribution of RGCs in intact, ONC + vehicle, ONC + mBM-MSCs and ONC + hBM-MSC retinas from the C57/BL6 strain. **(B)**: Bar graphs showing the total number of Brn3a^+^RGCs ± SD in syngeneic (top) and xenogeneic (bottom) transplanted retinas and their controls (*p < 0.05, non-parametric Mann-Whitney test). **(C)**: Representative isodensity maps (intact, and 3 and 5 days after ONC) or neighbour maps (90 days after ONC) showing the distribution of RGCs in intact, ONC + vehicle and ONC + mBM-MSCs retinas from the Balb/c strain. **(D)**: Bar graphs showing the total number of Brn3a^+^RGCs ± SD in allogeneic transplanted retinas and their controls. At the bottom of each map is shown the number of RGCs counted in the original retina. Colour codes for isodensity and neighbour maps appear in the first row. For more details see methods. *n* = 5 animals/group/time point at 3 and 5 days, and *n* = 4/group at 90 days.

**FIGURE 6:**
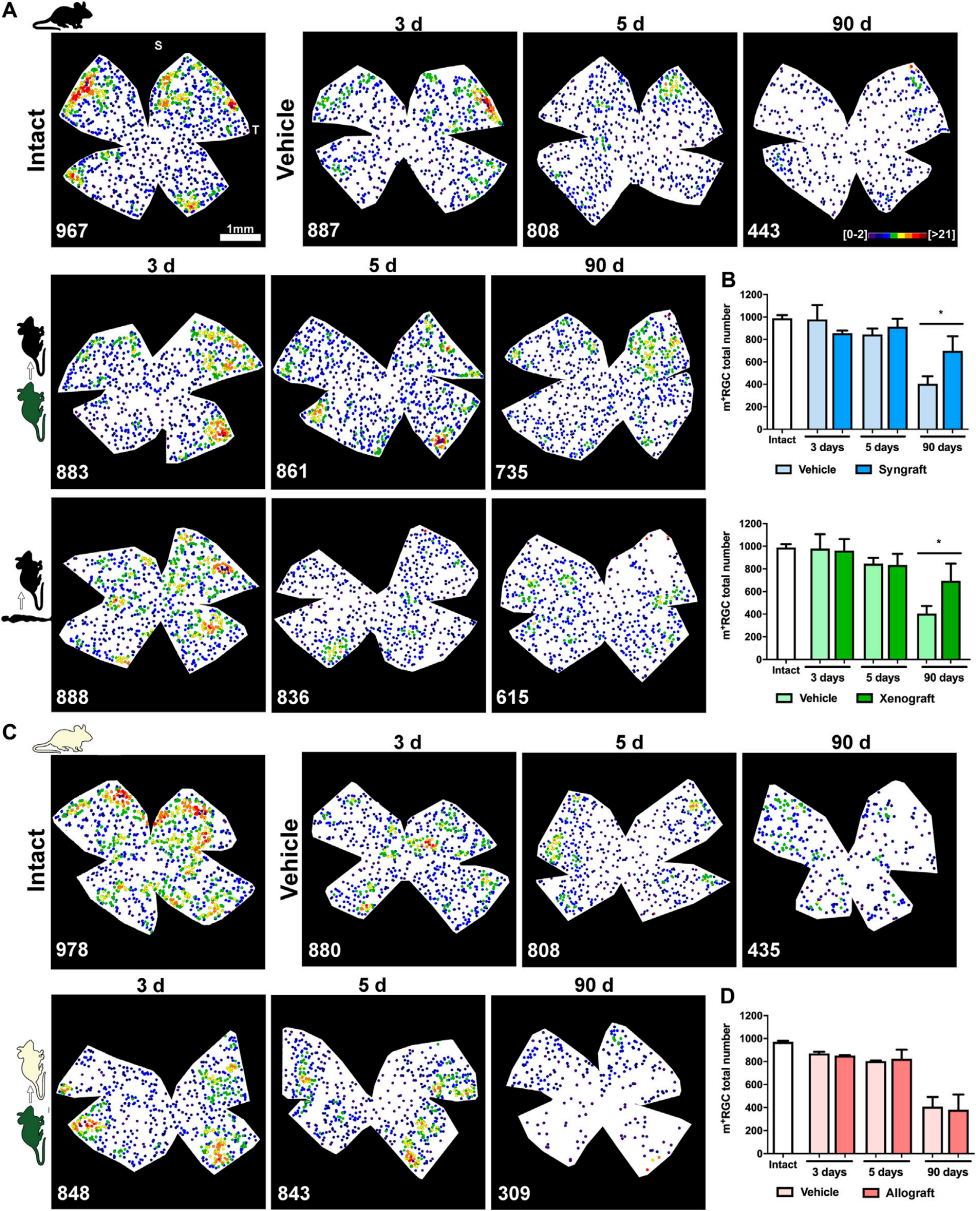
Syngeneic and xenogeneic transplants neuroprotect non-vision forming RGCs. **(A)**: Representative neighbour maps showing the distribution of m^+^RGCs in intact, ONC + vehicle, ONC + mBM-MSCs and ONC + hBM-MSC retinas from the C57/Bl6 strain. **(B)**: Bar graphs showing the total number of m^+^RGCs ± SD in syngeneic (top) and xenogeneic (bottom) transplanted retinas and their controls (*p < 0.05, non-parametric Mann-Whitney test) **(C)**: Representative neighbour maps showing the distribution of m^+^RGCs in intact, ONC + vehicle and ONC + mBM-MSCs retinas from the Balb/c strain. **(D)**: Bar graphs showing the total number of m^+^RGCs ± SD in allogeneic transplanted retinas and their controls. At the bottom of each map is shown the number of m^+^RGCs counted in the original retina. Colour codes neighbour maps appear in the first row. For more details see methods. *n* = 5 animals/group/time point at 3 and 5 days, and *n* = 4/group at 90 days.

In vehicle-treated retinas both functional subtypes of RGCs, vision-forming (Brn3a^+^) and non-vision forming (M1-M3 melanopsin^+^), underwent the course of axotomy-induced degeneration already reported (Valiente-Soriano et al., 2014; Sánchez-Migallón et al., 2018a) (Figure 5, Figure 6, and Supplementary Figure S1). Thus, Brn3a^+^RGC loss was significant at 3 days and progressed steadily up to 90 days when <1% of the original population remained (Figure 5), while 40% of m^+^RGCs that are more resilient to this injury (Sánchez-Migallón et al., 2018a), survived at 90 days (Figure 6).

The syngrafts had a small but significant neuroprotective effect on Brn3a^+^RGCs at 5 and 90 days (Figure 5B), a rescue that was not observed in the xenografted (Figure 5B) or allografted (Figure 5C) retinas. For m^+^RGCs, both the syngeneic and the xenogeneic transplants were beneficial, surviving 70% of their original population at 90 days (Figures 6A–D).

### Regenerating Axons Distant From the Lesion Site Are Observed Only in Syngrafted Retinas

**FIGURE 7:**
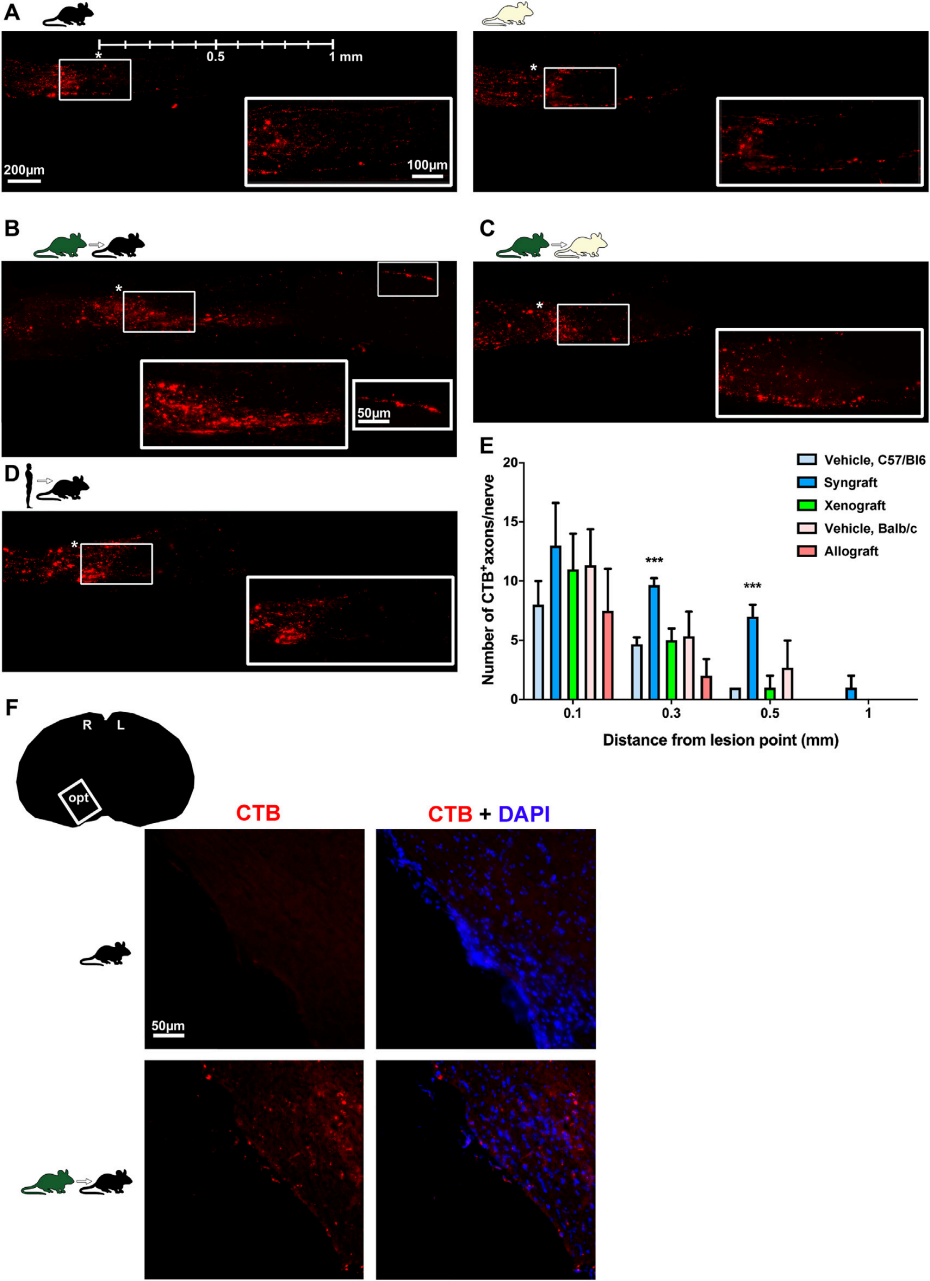
Syngeneic transplant supports axonal regeneration. **(A–D)** Photomontages of cleared optic nerves showing CTB-anterogradely traced RGC axons 90 days after ONC in vehicle-treated retinas, and BM-MSC-transplanted retinas. magnifications on the bottom right are from the framed areas. Lesion site is marked with an asterisk. Rostral left, caudal, right. *n* = 3 nerves/group. **(E)**: Mean number±SD of CTB^+^axons quantified at increasing distances from the lesion site (***p < 0.001 Kruskal-Wallis test, Dunn’s multiple comparisons test). **(F)**: CTB^+^axons in the optic tract were observed in one animal from the syngeneic group (*n* = 3 brains/group).

CTB-labelled axons were counted at increasing distances from the lesion site on cleared nerves (Figures 7A–E). Axonal regeneration was modest, but significantly higher in terms on number and distance in the syngeneic group. CTB-labelled axons were observed in the optic tract of one of the syngrafted animals (Figure 7F).

### Retinal Function Decreases After Axotomy and Is Further Impaired by the Xenograft

**FIGURE 8:**
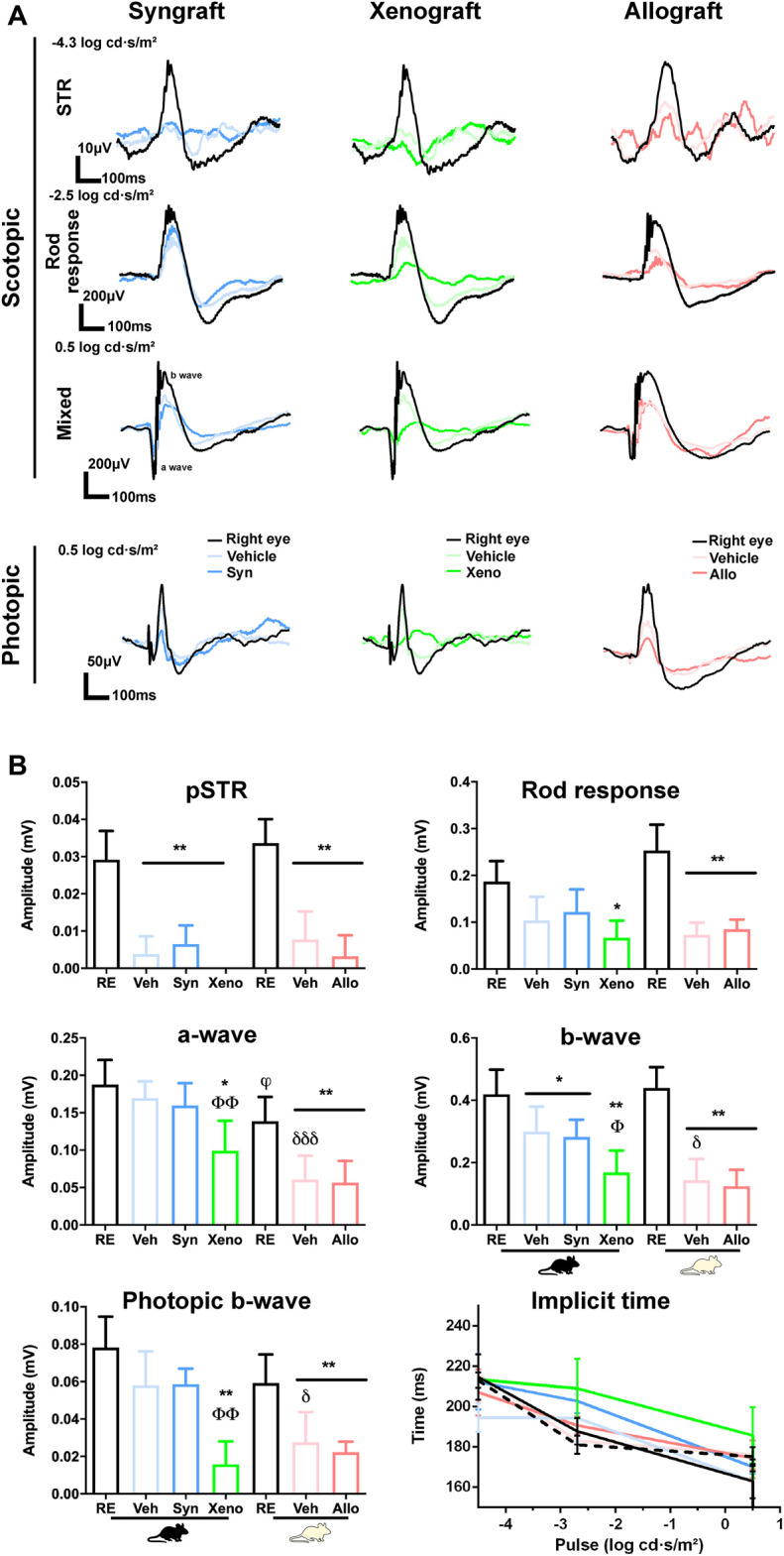
Retinal functionality decreases by axotomy and is further impaired by the xenograft. Electroretinographic analysis 90 days after ONC and vehicle or BM-MSCs intravitreal administration. Right eyes were used as controls. **(A)**: ERG traces in scotopic and photopic conditions. **(B)**: Graphs showing the quantification of the positive scotopic threshold response (pSTR, RGCs), scotopic rod response (rod bipolar cells), scotopic mixed response (b-wave: cone and rod bipolar cells; a-wave: cones and rods), photopic b-wave (cone bipolar cells), and implicit time at the different light pulses. Implicit time: lines colour-coded as graphs (dashed black line: Balb/c, solid black line, C57/Bl6). RE: right eyes. Veh: ONC + vehicle. Syn: ONC + syngraft. Xeno: ONC + xenograft. Allo: ONC + allograft. Mouse silhouettes represent the recipient strain. *Experimental vs. right eyes (*p < 0.05, **p < 0.01 Kruskal-Wallis test, Dunn’s multiple comparisons test). ^θ^Xenografted vs. ONC + vehicle (^θθ^ *p* < 0.01, non-parametric Mann-Whitney test), ^φ^albino *vs* pigmented right eyes (^φ^ *p* < 0.05, non-parametric Mann-Whitney test), ^δ^Albino vs. pigmented ONC + vehicle eyes (^δ^ *p* < 0.05; ^δδδ^ *p* < 0.001, non-parametric Mann-Whitney test). 4 animals/group were recorded.

We recorded retinal function at the end of the experiment (Figure 8A). The positive scotopic threshold response (pSTR), that measures RGC function, significantly decreased in all experimental retinas as expected, with no differences among groups (Figure 8B).

Regarding the rest of the ERG waves, which are related to photoreceptors and their bipolar cells, the most drastic effect was observed in the xenografted group. While in syngrafted and allografted retinas the decrease of functionality was similar to their vehicle controls, the xenograft caused a higher loss of function than its vehicle (Figure 8B), reaching significance for the a-wave (cones and rods), b-wave (cone and rod bipolar cells) and photopic b-wave (cone bipolar cells). No significant changes were observed in the implicit time (b-wave) of any group, although the response in xenografted animals was delayed in some pulses.

Finally, the functional impairment observed in axotomized albino retinas (vehicle group) was always higher than in the pigmented strain, reaching significance for the mixed response and the photopic b-wave (Figure 8B) (Alarcón-Martínez et al., 2010).

## Discussion

Treatment of neurodegenerative diseases is one of the major challenges of regenerative medicine. These are pleiotropic pathologies, from their cause to their physiological, cellular, and molecular signatures. Therefore, it seems unlikely to find a common denominator to target pharmacologically or genetically, even when narrowing by disease, because in most conditions there are a multitude of cell types affected.

Stem cells are a living medicine and produce bioactive molecules that vary according to the context in which they are grafted (Millán-Rivero et al., 2018). That is why stem cells are an exciting therapeutic avenue in neuroscience. What is needed to make stem cell therapy successful? i) cells should not induce a host response that makes them susceptible to being immunologically rejected; ii) grafted cells should remain alive long enough to rescue the compromised neurons; iii) target neurons should respond to the grafted cells, or, in other words, grafted cells must be the appropriate ones for each pathology and target cell type to obtain a beneficial therapeutic response. For cells to reach the clinic, they should be easily obtained, free of ethical concerns, and expandable *in vitro* without losing their properties. MSCs comply with these requirements.

Even then, treating patients with MSCs is difficult and clinical trials, mainly in phase III, are not being as successful as expected. There are many variables that need to be thoroughly investigated: i) are MSCs from different species the same? No. Even thought there are some similarities between human and mouse BM-MSCs (Jones and Schäfer, 2015), there are also differences in their secretomes (Harris, 1991); ii) do MSCs from the same species have the same properties? No, MSCs isolated from different tissues (Heo et al., 2016; Valencia et al., 2016; Grégoire et al., 2019), or from the same tissue but from different developmental stages (Gaetani et al., 2018), or healthy or diseased donors (Collins et al., 2014), or transplanted into different environments (Millán-Rivero et al., 2018) behave differently; iii) are preclinical studies comparable in terms of model, MSC type, and cell manufacture? are the ongoing clinical trials for CNS diseases homogeneous? again, the answer to both questions is no (Galipeau and Sensébé, 2018; Cui et al., 2019; Staff et al., 2019); iv) do the donor and host have an input in the therapeutic outcome? yes, as we have shown here, but again, to this we could add that different tissues from the same host may elicit a different response to the same MSC type.

MSCs are known for their immunomodulating properties, and secretion of paracrine factors (Khan and Newsome, 2019) both properties may be part of the therapeutic effect observed here and in other works using as model the injured retina (Zaverucha-do-Valle et al., 2011; Lucas-Ruiz et al., 2019a; Mesentier-Louro et al., 2019; Wen et al., 2021) or spinal cord (Ankeny et al., 2004; Wright et al., 2011. Reviewed in; Staff et al., 2019).

Our work agrees with previous reports showing in rats that the syngeneic transplant of BM-MSCs, enhances RGC survival and regeneration after optic nerve axotomy (Zaverucha-do-Valle et al., 2011; Mesentier-Louro et al., 2019). Our data in mouse extend further and we show that syngrafted BM-MSCs are able to rescue the two functional subtypes of RGCs, which are identified by their selective expression of Brn3a or melanopsin (Galindo-Romero et al., 2013a; Valiente-Soriano et al., 2014). Brn3a^+^RGCs are those sending visual-forming information to the brain, and it has been known for a while that they are more vulnerable to injury that melanopsin^+^RGCs, responsible for sending non-visual information (DeParis et al., 2012; González Fleitas et al., 2015; Nadal-Nicolás et al., 2015; Valiente-Soriano et al., 2015; Rovere et al., 2016; Vidal-Sanz et al., 2017; Sánchez-Migallón et al., 2018a). Thus, in this model the syngeneic transplant of BM-MSCs works in two different species, mouse and rat. It is therefore, tempting to hypothesise that the autologous transplant of BM-MSCs will have a positive effect on human patients with optic neuropathies.

Xenotransplants are used in preclinical models to test therapeutic effects of human cells in animals prior translation into clinic. Works from our lab (Millán-Rivero et al., 2018) and others (Wen et al., 2021) show that even when human cells neuroprotect, they also trigger an immune response that alters the host anatomically and functionally, as we show here as well. Neuroprotection using human cells seems to be dependent on the type of MSCs and neurons: hWJ-MSCs (perinatal MSCs isolated from the umbilical cord Wharton’s jelly) rescue Brn3a^+^RGCs (Millán-Rivero et al., 2018; Wen et al., 2021) while here we show that hBM-MSCs do not rescue them but do rescue the other functional subtype, m^+^RGCs.

In clinic, autologous transplants are preferable to allotransplants to avoid rejection and to increase the survival of the graft (Eliopoulos et al., 2005; Swanger et al., 2005). Here, we detected human and murine BM-MSCs in retinas at 3 and 5 days but not at 90 days. Therefore, BM-MSCs do not survive long-term irrespectively of the type of transplant. This may not be much of a problem because it has been proposed that the therapeutic effect of MSCs goes through a “hit and run” mechanism (Ankrum et al., 2014)**.** Our data agree with this mechanism, because neuroprotection and axonal regeneration was observed at 90 days, even though the grafts had already disappeared.

In our model, BM-MSC syngrafts are better than allografts, the latter having no impact on the variables evaluated here. However, the scenario in the clinic is not so straightforward because it has been shown that MSCs from patients with some pathologies have an altered gene expression profile and an impaired immunomodulatory/immunosuppressive activity and stemness compared to those obtained from healthy individuals (de Oliveira et al., 2015; Alicka et al., 2019). Therefore, for patients allotransplants with cells from healthy subjects may be a better option than autografts.

Finally, there is still much research to be done to successfully translate MSC therapy to the clinic. Research should focus on isolating specific variables, as we have done here comparing the effect of the three transplantation modalities on the same injury model. There are plenty of variables to study, as abovementioned, for example the impact of the donor or the recipient. In our previous work (Millán-Rivero et al., 2018), we tested human Wharton’s jelly MSCs isolated from *n* = 3 different umbilical cords, and the elicited neuroprotective effect was similar between them. In this case, the recipients were albino Sprague Dawley rats, and the human donors were different. In the clinic, patients are genetically different, except in syngeneic transplant between identical twins. Therefore, it would be very valuable to know whether the genetic background of different individuals (i.e., mice or rats of different strains) impacts the therapeutic potential of a given MSC type on a given disease model.

## Conclusion

This is the first study comparing the effect of the transplant type on the damaged central nervous system, using as model the axotomy of the optic nerve. Our results show that the syngeneic transplant of BM-MSCs rescues injured RGCs and promotes their regenerative capacity. Allogeneic transplantation has neither a positive nor a negative effect on the parameters measured here. The xenograft has a beneficial effect on non-vision forming RGCs but not on vision forming-RGCs, indicating that the future of MSC treatments may have to be tailored not only to the disease but also to the neuronal type. Finally, the xenotransplant induces pathological changes in the host retina, and a decrease in functionality compared to the untreated groups. Therefore, because the host response probably has an important effect on the therapeutic outcome, results of human cells in animals should be interpreted with caution.

## Data Availability

The original contributions presented in the study are included in the article/Supplementary Material, further inquiries can be directed to the corresponding author.

## Abbreviations

ATMP, advanced therapy medical product; BAF, blue autofluorescence filter; BM-MSC, bone marrow mesenchymal stromal cells; CTB, β subunit of the cholera toxin; DMEM, Dulbecco Modified Eagle Medium; ERG, electroretinography; FBS, fetal bovine serum; GFP, green fluorescent protein; hBM, human bone marrow; mBM, mouse bone marrow; MSC, mesenchymal stromal cells; OCT, optical coherence tomography; PBS, phosphate buffered saline; P/S, penicillin/streptomycin; R/T, room temperature; RGC, retinal ganglion cell; SD: standard deviation.
