# Supplementary figures and images for: Neuroprotection and Axonal Regeneration Induced by Bone Marrow Mesenchymal Stromal Cells Depend on the Type of Transplant
Source: Front Cell Dev Biol. 2021 Nov 4;9:772223. doi: 10.3389/fcell.2021.772223 (PMC8600074; doi:10.3389/fcell.2021.772223)

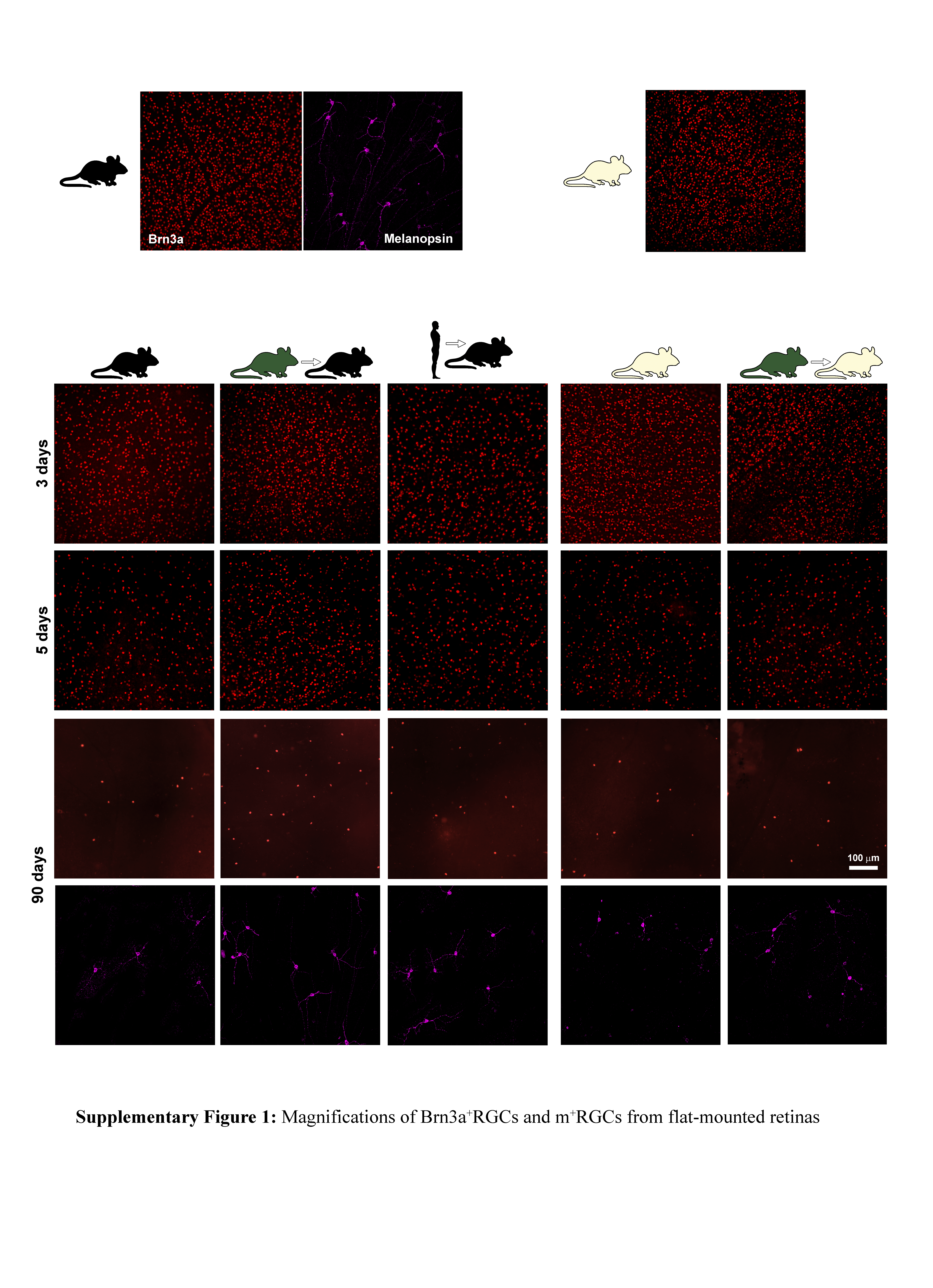

Supplement: Supplementary file 1 [file Image1.TIF]
